# Patients' Perception of Bone and Tissue Excision, and the Size and Weight of Prostheses at Total Knee Arthroplasty

**DOI:** 10.1055/s-0037-1604010

**Published:** 2017-07-13

**Authors:** Benjamin Rossi, Narlaka Jayasekera, Fionnuala Anne Kelly, Keith Eyres

**Affiliations:** 1 Knee Reconstruction Unit, Princess Elizabeth Orthopaedic Centre, Royal Devon and Exeter Hospital, Exeter, United Kingdom; 2 Department of Orthopaedics, Gold Coast University Hospital, Gold Coast, Australia

**Keywords:** knee arthroplasty, prostheses, bone excision, patients' perception

## Abstract

The aim of this study is to ascertain patients' perception of the amount of bone and tissue excision and size and weight of their implanted prostheses at total knee arthroplasty (TKA). To our knowledge, no prior study in the English orthopaedic literature has analyzed these parameters against patient perception of TKA. In a prospective study of eight consecutive TKA (six primary and two single-stage revision TKA procedures) by a single surgeon, patients estimated the weight of their implanted knee. We assessed actual weights of their implants and bone cement. Patients estimated the size of their prostheses by sketching the tibial and femoral bone cuts upon a printout of an anteroposterior and lateral radiographs of their preoperative knee. We utilized an articulated plastic model knee for patient reference. Our study shows almost half a kilogram of weight is added postoperatively to the surgical site as a result of tissue excision, explanted material, and implanted prosthesis and cement. All patients overestimated the weight of their implanted prostheses and extent of bone excision. Thus, even ‘well-informed’ patients overestimate their bone resection and weight of implanted prosthesis at TKA. We postulate such misconceptions among TKA patients are common, and may impact negatively upon patient perception of TKA, their postoperative recovery and outcome.

Patients undergoing total knee arthroplasty (TKA) may not accurately perceive the true nature of the procedure in terms of extent of host tissue excision and weight and size of the implanted knee. Such misconceptions may influence a patient's decision to proceed to TKA and also impact upon their operative outcomes. Several articles describe weight gain in patients after TKA, 1 2 3 yet, to our knowledge, only one study has thus far quantified the difference in weight between excised host tissue, and newly implanted TKA and bone cement. 1

The aim of this study was to ascertain patients' understanding of their TKA with particular reference to their perception of the amount of bone and soft tissue excised, and the true weight of their implanted prostheses and bone cement. To our knowledge, no prior study in the English orthopaedic literature has analyzed these parameters together with patients' perception of TKA.

## Methods

We undertook a prospective analysis of eight consecutive patients undergoing TKA by a single surgeon (K.E.) at a high-volume knee arthroplasty unit in a tertiary referral center for hip and knee disorders in the United Kingdom. Six primary (Triathlon CR, Stryker Orthopaedics) and two single-stage revision TKA procedures (one medial unicompartmental arthroplasty revised to a Triathlon CR and one TKA revised to a LINK Endo-Model hinge knee prosthesis, Waldemar LINK GmbH & Co. KG) were included in the study.

All elective TKA patients at our institution attend a nurse-led preoperative assessment clinic where patients are provided information and counseled for surgery. They receive an information booklet (“Joint Pathway in partnership with Royal Devon and Exeter NHS Foundation Trust, patient's guide for knees before, during, and after replacement,” Stryker 2012) and a digital video disc (DVD) “A patient's guide to Knee & Hip Replacement,” Stryker. This information includes animation of a TKA procedure depicting the extent of bone resection at the femur and tibia. It also shows the relative size of implanted knee. We asked all patients whether they received the booklet and DVD, and whether they used these resources prior to surgery.

We then asked patients to estimate the weight of their implanted knee (in their chosen units, i.e., pounds/kilograms) and compared this to the actual weights of their implants. We also asked patients to estimate the size of their implanted prostheses. This assessment was made by the patient sketching two lines to depict the tibial and femoral bone cuts upon an A4 size printout of anteroposterior and lateral radiograph of their preoperative knee, with an articulated plastic model knee for reference.

**Fig. 1:**
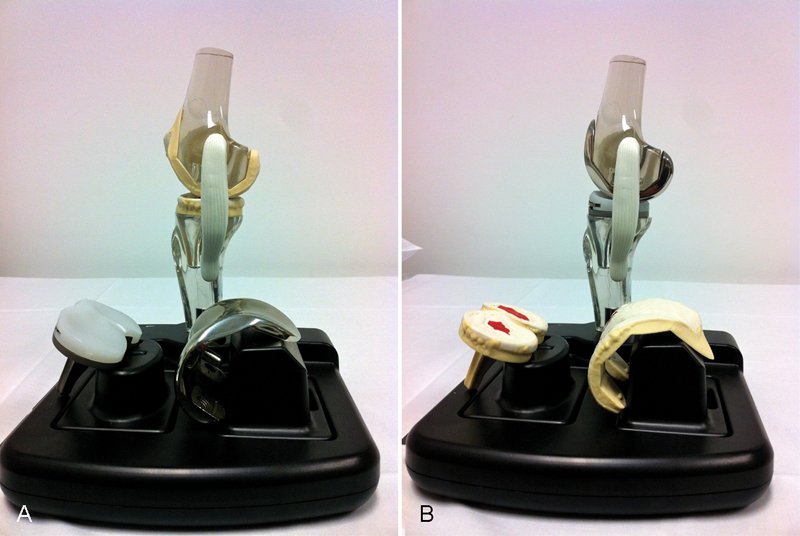
( **A** ) Articulated transparent polycarbonate model of the knee. ( **B** ) Depicts the same model of the knee with assembled cruciate-retaining primary knee prostheses of true weight.

Once these questions had been completed, we informed each patient of the true weight differential gained with their implant and also showed them their postoperative radiograph and a reference model of an articulated plastic knee depicting the cut femoral and tibial bone surfaces ( Fig. 1A, B ).

The weight data were collected by weighing the implants complete with packaging and then subtracting all packaging weight and adding the weight of the cement implanted; then we subtracted the weight of the tissue ± explanted prostheses and bone cement was removed to obtain a total weight differential. These data were gathered prospectively by investigator (N.J.) using a digital weighing scale (model 1035 SSBKDR, Salter, HoMedics Group Ltd). Implant size was measured on postoperative radiographs with the use of the measurement tool on our patient archiving system (GE Healthcare).

## Results

Mean weight estimate by the patient was 972.25 g, mean actual weight was 388.38 g, mean tibial size estimate 37.80 mm, mean femur size estimate 47.67 mm, mean actual tibial size 10.84 mm, and mean actual femur size 20.89 mm ( Table 1 ).

**Table 1:** Patient data

Patient	1	2	3	4	5	6	7	8
**Booklet**	Y	Y	N	Y	Y	Y	Y	Y
**DVD**	Y	Y	N	Y	Y	Y	Y	Y
**Used booklet**	Y	Y	N	Y	Y	Y	Y	Y
**Used DVD**	N	N	N	N	Y	Y	N	Y
**Tibia estimate (mm)**	46.15	110.67	9.90	57.64	9.02	7.06	45.44	16.48
**Femur estimate (mm)**	87.73	99.86	31.58	77.28	8.55	16.91	44.48	15.00
**Weight estimate (g)**	907	750	2721	453	907	680	453	907
**Tibia actual (mm)**	12.35	9.82	9.22	9.23	11.2	13.69	10.27	10.91
**Femur actual (mm)**	12.57	13.48	21.93	28.79	32.3	33.75	12.80	11.48
**Actual weight (g)**	529	469	478	525	751	405	433	473
**Implant type**	PT	PT	RT	PT	RL	PT	PT	PNFT

Abbreviations: DVD, digital video disc; L, LINK Endo-Model; NFT, nickel-free Triathlon; P, primary; R, revision; T, Triathlon.

## Discussion

This study shows that patients' perception of their operation varied widely, mostly, we feel, in a negative way. By overestimating the weight of implant, they believed they had undergone greater bone resection and subsequently gained more weight than they actually had. We feel this may negatively impact upon their postoperative recovery. Gain in patient weight following TKA has been confirmed by previous authors, 1 2 3 but ours and that of Lee et al 4 are unique, in the weight analysis of explanted host tissue and newly implanted prosthesis and bone cement. Our study also included the analysis of two patients undergoing revision total knee replacement. Our findings do concur with Lee et al 4 in the magnitude of weight gained at the knee following TKA. The study by Lee et al, 4 however, was limited, as they did not analyze patients' perception of TKA. The change in patients' weight after TKA shown in previous studies 1 2 3 may be significantly influenced by fluid shifts during the perioperative period and thus not truly reflects the weight of explanted and implanted materials at TKA. Our study, however, exclusively analyzed this very aspect and has shown that almost half a kilogram of weight is added postoperatively to the surgical site as a direct consequence of tissue excision, explanted material, and implanted prosthesis and cement. This is especially pertinent to the study by Abu-Rajab et al, 3 which shows a median weight gain postoperatively of 500 g, which according to our study, may have possibly been due to the implanted components alone.

Our study shows TKA patients perceive exaggeration in their bone and soft tissue excision, which may result in their inference of having more extensive surgery and heavier prostheses implanted within their knee. We postulate that this may in turn unfavorably skew their perception of their TKA and likely negatively impact upon their postoperative recovery and rehabilitation.

We found poor utility of the DVD provided to TKA patients at preoperative assessment clinic with only three of the eight patients viewing the video. This may risk a less than well-informed patient at point of the procedure. We, therefore, suggest it unwise for clinicians to assume patients would routinely utilize such resources made available. It may be that patients would be better informed through a designated patient group TKA DVD screening session or possibly a loop screening of the DVD in the reception area as patients await their appointment. All patients believed their participation in this study had a positive impact upon their outlook and recovery.

The positive feedback from all eight patients in our study suggests that preoperative visual demonstration and explanation of TKA utilizing an articulated model knee, which demonstrates the planned proximal tibial and distal femoral resections and the extent of the implanted components ( Fig. 1A, B ) may be a more effective way to correctly inform patients of their planned procedure during preoperative education and assessment. A knee model with true weighted prostheses, which the patient may handle, may be particularly helpful in achieving this.

Limitations of this study include its small sample size and mixture of primary and revision TKA procedures and the absence of patient-related outcome measures. A larger randomized study may highlight the impact of patient education and perception of TKA more definitively. We also acknowledge that our demonstrated weight differences may vary in their impact upon individual patients, possibly influencing lighter patients more significantly. This aspect was not assessable within remit of this study. The variability of pre- and postoperative radiographic projections, which made measurement somewhat difficult; therefore, we chose independent measurement by two clinicians blinded to each other's assessment. We found no disparity between their assessments. Despite these limitations, we feel we have adequately demonstrated clear and significant misconception of the TKA procedure among most patients previously considered as well informed using a to date acceptable preoperative education and assessment process. We feel there would be significant improvement in patients' understanding with the use of an articulated model knee demonstrating femoral and tibial bone cuts with matching detachable weighted tibial and femoral implant models.

We believe further investigation is warranted to look at these important, yet largely under, investigated aspects of TKA and the impact upon patient outcome.
